# Both Hypo-Connectivity and Hyper-Connectivity of the Insular Subregions Associated With Severity in Children With Autism Spectrum Disorders

**DOI:** 10.3389/fnins.2018.00234

**Published:** 2018-04-11

**Authors:** Jinping Xu, Hongwei Wang, Lu Zhang, Ziyun Xu, Tian Li, Zhifeng Zhou, Zhenhui Zhou, Yungen Gan, Qingmao Hu

**Affiliations:** ^1^Institute of Biomedical and Health Engineering, Shenzhen Institutes of Advanced Technology, Chinese Academy of Sciences, Shenzhen, China; ^2^Department of Radiology, Shenzhen Children's Hospital, Shenzhen, China; ^3^Graduate School of Education, Peking University, Beijing, China; ^4^Psychological Department, Shenzhen Children's Hospital, Shenzhen, China

**Keywords:** autism spectrum disorders, hypo-connectivity, hyper-connectivity, insula, brainnetome atlas

## Abstract

Some studies identified hypo-connectivity, while others showed hyper-connectivity of the insula in the autism spectrum disorders (ASD). These contradictory findings leave open the question of whether and to what extent functional connectivity of the insula is altered and how functional connectivity of the insula is associated with the severity of ASD. A newly emerging insular atlas that comprises multiple functionally differentiated subregions provides a new framework to interpret the functional significance of insular findings and uncover the mechanisms underlying the severity of ASD. Using the new insular atlas, the present study aimed to investigate the distinct functional connectivity of the insular subregions and their associations with ASD severity in a cohort of 49 children with ASD and 33 typically developing (TD) subjects. We found that compared with TD group, the ASD group showed different connectivity patterns in the left ventral agranular insula, right ventral dysgranular and granular insula, and dorsal dysgranular insula, characterized by significant hyper-connectivity and/or hypo-connectivity with special brain regions. Furthermore, both the hypo-connectivity and hyper-connectivity patterns of the insular subregions were significantly associated with the severity of ASD symptoms. Our research demonstrated distinct functional connectivity patterns of the insular subregions and emphasized the importance of the subdivisions within the insula to the potential impact of functional difference in children with ASD. Moreover, these results might help us to better understand the mechanisms underlying the symptoms in children with ASD and might elucidate potential biomarkers for clinical applications.

## Introduction

Autism spectrum disorders (ASD) are prevalent neurodevelopmental disorders characterized by deficits in social interaction; verbal and nonverbal communication; and restricted and stereotyped patterns of behavior, interests, and activities (Minshew and Williams, 2007). Although previous studies have directly linked aberrant intrinsic brain connectivity in ASD to specific symptoms, such as poor social functioning, severe restricted, and repetitive behaviors (Monk et al., 2009; Weng et al., 2010), the exact mechanisms underlying these symptoms are poorly understood. To date, broad brain regions were thought to specially contribute to ASD symptoms. The insula is one such brain regions with the suggestion that the atypical functional connectivity of the insula may be important in the neuropathology of ASD (Uddin and Menon, 2009; Uddin et al., 2013b). Specifically, it has been demonstrated that adolescents with ASD show decreased regional homogeneity in the right insula (Paakki et al., 2010). Functionally, a comprehensive meta-analysis of functional imaging studies has revealed hypo-activation in the right anterior insula during various social tasks in ASD (school-age children, adolescents, and adults) (Di Martino et al., 2009). Regarding functional connectivity, reduced functional connectivity of the anterior, middle, and posterior insula with specific brain regions involved in different brain networks was identified in adolescent and adult ASD (Ebisch et al., 2011; Von Dem Hagen et al., 2013; Di Martino et al., 2014). These results all confirmed the hypo-connectivity of the insula and strongly supported the hypo-connectivity theory of the ASD. However, accumulating evidence of brain hyper-connectivity also exists in the domains of visual processing, emotion processing, memory, and language in ASD (Noonan et al., 2009; Shih et al., 2010, 2011). Specifically, Uddin et al. (2013b) observed stronger functional connectivity of the insula in 20 children with ASD and replicated this finding in an independent cohort, suggesting that the hyper-connectivity of the salience network including the bilateral insula may be a distinguishing feature in children with ASD. Although Uddin et al. proposed that discrepancies between findings of ASD related hypo-connectivity and hyper-connectivity might be reconciled by taking developmental changes into account (Uddin et al., 2013a), it remains unclear whether the insular subregions may account for observed inconsistencies in ASD. It is noteworthy that the insula is comprised of separate subregions, only assessing it as a whole region may obscure individual differences of functional connectivity with insula in ASD.

The insula is reported to be involved in diverse functions, including gustatory and olfactory processing, components of somatosensation, interoception, motivation, and the maintenance of homeostasis (Critchley, 2005; Seminowicz and Davis, 2007; Craig, 2009). Moreover, the insula also showed left/right differences, which could be related to the hypothesis that the two side of the insula subserve different functions and are linked to different circuits (Craig, 2009; Cauda et al., 2012). For example, the right insula has been proposed as a key node between the default mode network (DMN) and the central executive/attentional network (Sridharan et al., 2008). In addition, other studies (Cauda et al., 2011, 2012) identified that the anterior part related to salience network was found to be frankly lateralized on the right and the visuomotor integration network (posterior cluster) found to have a mild right lateralization. Given these variable functions and laterality of the insula, multiple functionally differentiated subregions with distinct patterns of connectivity were identified in the insula using k-means clustering of insula voxels (Jakab et al., 2012; Kelly et al., 2012), structural connections (Cloutman et al., 2012), clustering of a priori instantiated regions of interest (Cauda et al., 2011), meta-analytic approaches (Kurth et al., 2010b; Cauda et al., 2012), clustering of resting state functional connectivity patterns (Deen et al., 2011; Chang et al., 2013; Gordon et al., 2016), dynamic functional network connectivity (Nomi et al., 2016), and anatomy connectivity patterns (Fan et al., 2016). Among all these atlas of the insula, the insular subregions in Brainnetome atlas has been not only well established to reflect functional segregation of the insula, but also related well to other functional and histological maps of the insular cortex (Kurth et al., 2010a; Kelly et al., 2012; Chang et al., 2013; Morey et al., 2013). Moreover, different connectional, functional connectivity patterns, and behavioral domains of insular subregions were identified and shown along with the atlas (http://atlas.brainnetome.org/bnatlas.html), suggesting the possibility that the insular subregions may differ in their vulnerability to the ASD and may play different roles in the core symptoms of ASD. Thus, investigating functional connectivity of insula using the brainnetome atlas in the ASD will provide further insights to better understand the mechanisms underlying the core symptoms of ASD and may lead to identifying potential biomarkers that could be used in clinical situations. In addition, this atlas was successfully used in a recent study, which showed disrupted functional connectivity patterns of the insular subregions involved in different neural circuits associated with the contrary impacts on the depressive symptoms in drug-free major depressive disorder (Wang et al., 2017).

Using the new insula atlas (Fan et al., 2016), the present study aimed to investigate the distinct functional abnormalities in each of the insular subregions in a cohort of 49 children with ASD and 33 typically developing (TD) subjects. To further examine the relationship between the functional connectivity of each subregion of the insula and the severity of ASD, correlations were calculated between scores on the Autism Diagnostic Observation Schedule (ADOS) and Autism Diagnostic Interview-Revised (ADI-R) and altered functional connectivity of insular subregions.

## Materials and methods

### Participants

We used the dataset of the University of California, Los Angeles, one of the subsamples in the Autism Brain Imaging Data Exchange database (http://preprocessed-connectomes-project.org/abide/download.html). In regards to inclusion criteria, ASD had a prior clinical diagnosis of autism based on criteria from the Diagnostic and Statistical Manual of Mental Disorders IV, which was confirmed with the ADOS (Lord et al., 2000) and/or ADI-R (Lord et al., 1994). The ADOS has subscores for social interaction (ADOS social) and communication (ADOS communication), which are combined into a total score (ADOS total). The ADI-R has subscores for social interaction (ADI-R social), verbal (ADI-R verbal), and repetive behaviors (ADI-R). TD participants had no history of any genetic, neurological, psychiatric, or developmental disorders. In addition, they could not have a first degree relative with an ASD diagnosis. Verbal, performance, and full-scale intelligence quotients (IQs) were assessed for each participant using the four subsets of the Wechsler Abbreviated Scale of Intelligence or the full Wechsler Intelligence Scale for Children (Wechsler, 1999). Handedness was assessed via parental reports on a questionnaire. This study was carried out in accordance with the recommendation of Institutional Review Board of University of California, Los Angeles with informed consent from all subjects. All subjects gave written informed consent in accordance with the Declaration of Helsinki. The protocol wad approved by the Institutional Review Board of University of California, Los Angeles.

The initial dataset includes 62 ASD and 47 TD individuals. Based on the criteria from the Quality Assessment Protocol (http://preprocessed-connectomes-project.org/quality-assessment-protocol/ and http://preprocessed-connectomes-project.org/abide/quality_assessment.html), we only included subjects whose functional quality are all ok after manual checking. Moreover, subjects whose mean frame-wise displacement (FD) is >1 mm was also excluded. Finally, the sample included 49 ASD (age ranged from 8 to 17) and 33 TD subjects (age ranged from 9 to 17) (Table 1). The two groups did not significantly differ based on age, gender, mean FD, full-scale, verbal, and performance IQs. In the final sample, ADOS and ADI-R scores were available for only 48 ASD patients. Of the subjects in the ASD group, 23 individuals reported the use of one or more psychotropic medications.

**Table 1:** Demographics and clinical characteristics.

**Characteristic**	**TD**	**ASD**	***p*-value**
Sample size	33	49	–
Gender (female/male)^a^	6/27	6/43	0.456
Handness (left/right)^a^	3/30	5/44	0.868
Mean FD^b^:mean ± SD	0.13 ± 0.18	0.19 ± 0.19	0.114
Age^b^:mean ± SD	13.30 ± 2.04	13.05 ± 2.46	0.639
Verbal IQ^b^:mean ± SD	105.21 ± 10.74	102.93 ± 13.66	0.424
Performance IQ^b^:mean ± SD	101.63 ± 10.58	100.30 ± 13.91	0.643
Full scale IQ^b^:mean ± SD	103.81 ± 9.56	101.42 ± 13.33	0.378
ADOS total:mean ± SD	–	10.66 ± 3.54 (*n* = 48)	–
ADOS communication:mean ± SD	–	3.16 ± 1.43 (*n* = 48)	–
ADOS social:mean ± SD	–	7.50 ± 2.45 (*n* = 48)	–
ADI-R social:mean ± SD	–	20.02 ± 5.33 (*n* = 48)	–
ADI-R verbal:mean ± SD	–	16.29 ± 4.65 (*n* = 48)	–
ADI-R repetitive behaviors: mean ± SD	–	7.14 ± 2.55 (*n* = 48)	–

a *The p-value was obtained by a chi-square test*.

b *The p-value was obtained by a two-tailed two-sample t-test; –, indicates no data available. TD, typically developing; ASD, autism spectrum disorders; FD, framewise displacement; IQ, intelligence quotients; ADOS, Autism Diagnostic Observation Schedule; and ADI-R, Autism Diagnostic Interview-Revised*.

### MRI data acquisition

All resting-state fMRI scans were acquired on a Siemens 3 T Trio at the University of California, Los Angeles. During data acquisition, subjects were asked to relax, keep their eyes open, and keep their head still. A white screen with a black fixation cross in the middle of the screen was presented. The T2-weighted functional images were collected with the following settings: repeat time = 3,000 ms, echo time = 28 ms, matrix size = 64 × 64, field of view = 192 mm, and thickness = 4 mm, no gap, interleaved acquisition, with an in-plane voxel dimension of 3 × 3 mm. The T1-weighted magnetization-prepared rapid gradient-echo images were collected with the following settings: repeat time = 2,300 ms, echo time = 2.84 ms, field of view = 256 mm, flip angle = 9°, and thickness = 1.2 mm, interleaved acquisition, with an in-plane voxel dimension of 1 × 1 mm.

### Resting-state fMRI data preprocessing

The fMRI data were preprocessed under the Preprocessed Connectomes Project (http://preprocessed-connectomes-project.org/) with the Data Processing Assistant for Resting-State fMRI (DPARSF, http://preprocessed-connectomes-project.org/abide/dparsf.html). For each participant, the preprocessing steps were as follows: (1) all volume slices were corrected for different signal acquisition times; (2) the time series of images for each subject were realigned using a six-parameter (rigid body) linear transformation; (3) individual structural images were co-registered to the mean functional image after realignment using a six degrees-of-freedom linear transformation without resampling; (4) the transformed structural images were then segmented into gray matter, white matter, and cerebrospinal fluid; (5) the Diffeomorphic Anatomical Registration Through Exponentiated Lie algebra (DARTEL) tool (Ashburner, 2007) was used to compute transformations from individual native space to MNI space; (6) the Friston 24-parameter model (Friston et al., 1996) was utilized to regress out head motion effects from the realigned data (Satterthwaite et al., 2013; Yan et al., 2013); (7) the white matter, cerebrospinal fluid, and global signals were regressed out; (8) linear and quadratic trends and temporal band pass filtering (0.01–0.1 Hz) were performed; (9) corresponding maps were then registered into MNI space with 3 mm three cubic voxels by using transformation information acquired from DARTEL; and (10) the maps were further smoothed by a kernel of 6 mm.

#### Definition of the insular subregions

The bilateral insula subregions were defined by the 50% probability maps in the Brainnetome Atlas. Six subregions of insula in each brain hemisphere were defined as seed areas, including the hypergranular insula, ventral agranular insula, dorsal agranular insula, ventral dysgranular and granular insula, dorsal granular insula, and dorsal dysgranular insula. For resting-state functional connectivity (RSFC) analyses, the insula subregions were resampled into 3 × 3 × 3 mm^3^ in MNI space.

### The whole brain RSFC patterns in the ASD and TD groups

For all the subjects, the RSFC was defined by Pearson correlation coefficients between the mean time series of each seed region and that of each voxel in the rest of the brain. We used the binary gray matter mask in SPM before computing the whole brain RSFC. Correlation coefficients were converted to *z*-values using Fisher's *z* transformation to improve normality. Next, one-sample *t*-test was performed to identify voxels which showed significantly positive or negative correlations with the seed region in these normalized correlation maps. For all the above voxel-wise comparisons, significance was determined with a voxel-level corrected threshold of *p* < 0.001 and a cluster-level corrected threshold of *p* < 0.05 using the Gaussian random field (GRF) correction in the DPABI (http://rfmri.org/dpabi).

### Altered RSFC of the insular subregions in ASD

First, two-sample *t*-tests were implemented using DPABI to map group difference of RSFC between ASD and TD groups with the age, gender, handedness, verbal IQs, performance IQs, and full-scale IQs as covariates. Significance was determined with a voxel-level corrected threshold of *p* < 0.001 and a cluster-level corrected threshold of *p* < 0.05 using the GRF correction in the DPABI.

Then, we calculated the mean RSFC of the regions which showed significantly altered RSFC with subregions of the insula in the ASD and TD group. To exclude the effects of global signal, we re-analyzed the mean RSFC using rs-fMRI data with global signal.

### Correlation analyses between the RSFC and the severity of ASD

Finally, the partial correlation analyses between the average *z*-score of the region (RSFC) and the severity scores of ASD (ADOS and ADI-R scores) were performed in the ASD group with the age, gender, handedness, verbal IQs, performance IQs, and full-scale IQs as covariates using SPSS. The statistical level with *p* < 0.05 was considered as significant.

## Results

### Distinct RSFC patterns of insular subregions between the ASD and TD groups

**Figure 1:**
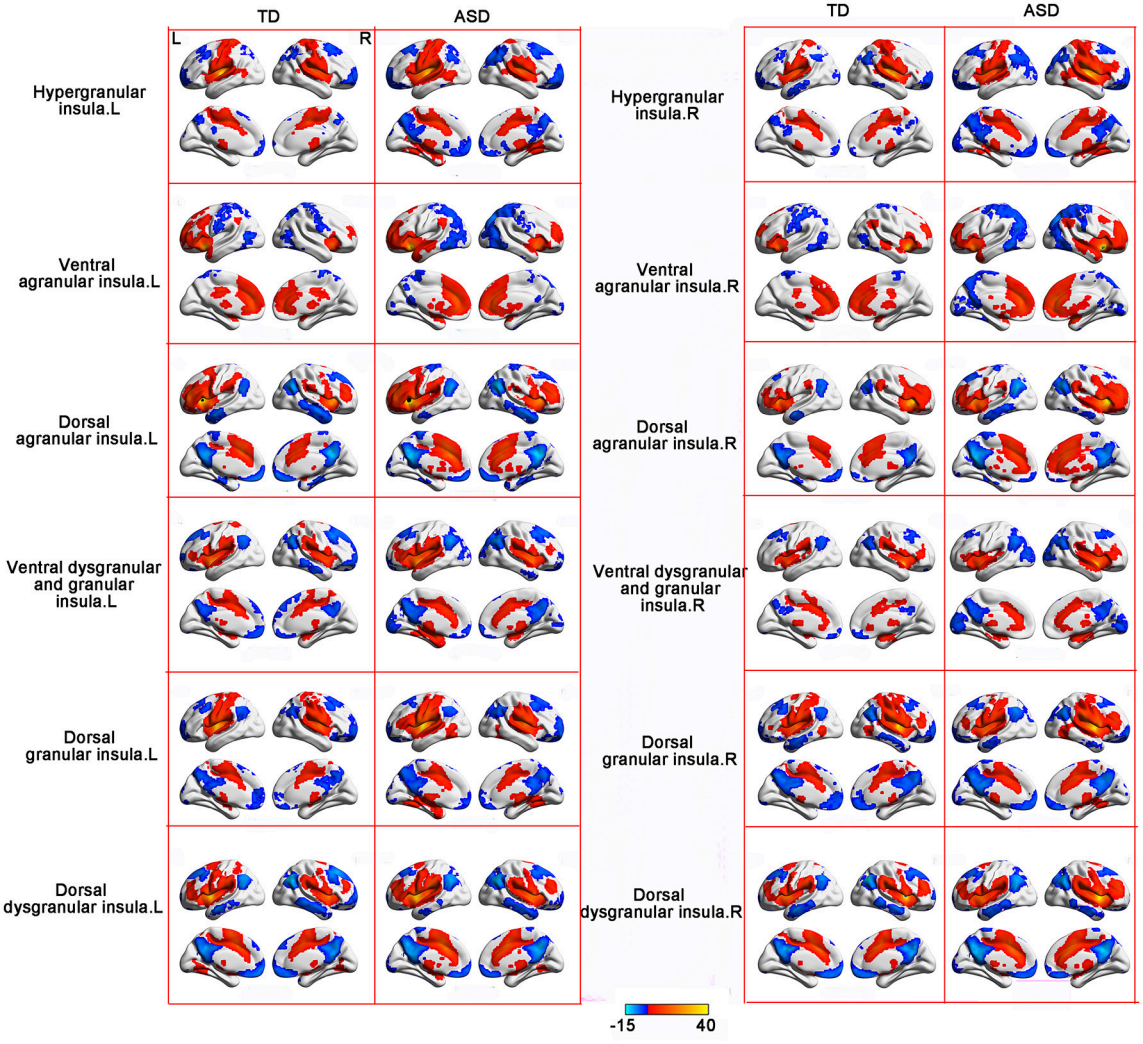
RSFC patterns of the insular subregions in the ASD and TD groups. One sample *t*-tests were used to identify the group statistical maps. The significance level was determined with a voxel-level corrected threshold of *p* < 0.001 and a cluster-level corrected threshold of *p* < 0.05 using the Gaussian random field (GRF) corrections. The red and blue colors represent positive and negative functional connectivity with the seed regions.

**Figure 2:**
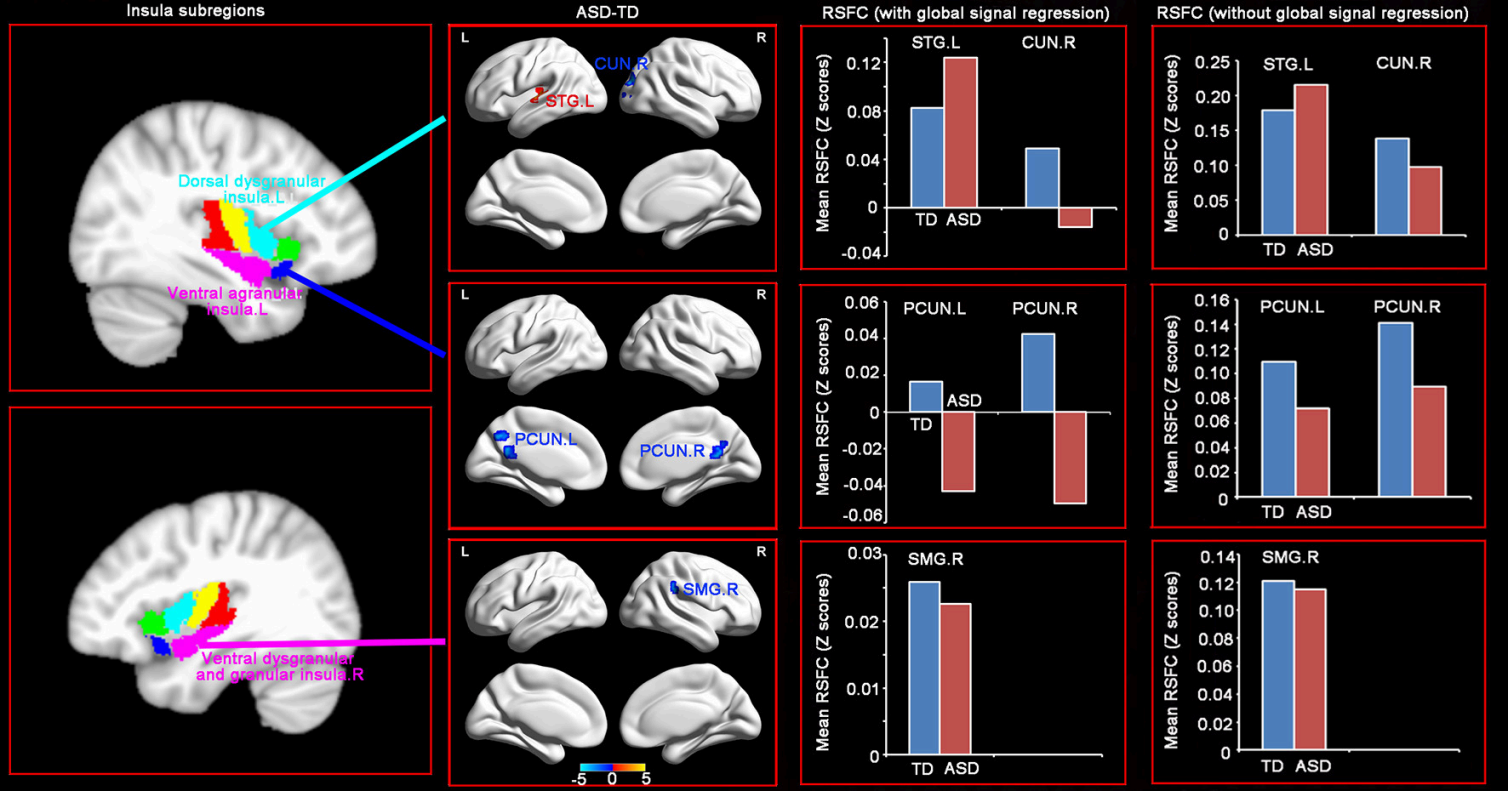
Distinct RSFC patterns of the insular subregions in ASD. Two sample *t*-tests were used to identify the significant differences in functional connectivity between the ASD and TD groups with the age, gender, handedness, verbal IQs, performance IQs, and full-scale IQs as covariates. The significance was determined with a voxel-level corrected threshold of *p* < 0.001 and a cluster-level corrected threshold of *p* < 0.05 using the Gaussian random field (GRF) corrections. The red and blue colors represent increased and decreased functional connectivity respectively in the ASD group compared with the TD group. The results of mean RSFC with and without global signal showed similar patterns between the two groups.

RSFC analyses based on the insular subregions resulted in distinct connectivity maps in the TD and ASD groups (Figure 1). Statistical comparisons between these maps showed significant differences of RSFC between the TD and ASD groups in the left ventral agranular insula, right ventral dysgranular and granular insula, and left dorsal dysgranular insula (Figure 2 and Table 2). Specifically, children with ASD showed hypo-connectivity between the left ventral agranular insula and the bilateral precuneus (PCUN), between ventral dysgranular and granular insula and the right supramarginal gyrus (SMG.R), and between the left dorsal dysgranular insula and the right cuneus (CUN.R). Moreover, children with ASD also showed hyper-connectivity between the left dorsal dysgranular insula and the left superior temporal gyrus (STG.L).

**Table 2:** Brain regions showing significant difference of functional connectivity with the insular subregions.

**Seed regions**	**Abnormal regions**	**Types of connectivity**	**Number of voxels**	**Peak intensity**	**Peak coordinates**		
Left ventral agranular insula	The right precuneus	Hypo-connectivity	46	−4.316	12	−56	31
	The left precuneus	Hypo-connectivity	36	−4.46	−12	−59	43
Right ventral dysgranular and granular insula	The right supramarginal gyrus	Hypo-connectivity	28	−4.1961	45	−32	31
Right dorsal dysgranular insula	The left superior temporal gyrus	Hyper-connectivity	46	4.8826	−48	−23	4
	The right cuneus	Hypo-connectivity	58	−4.1796	21	−86	4

### The relationship between RSFC and the severity of ASD in children

**Figure 3:**
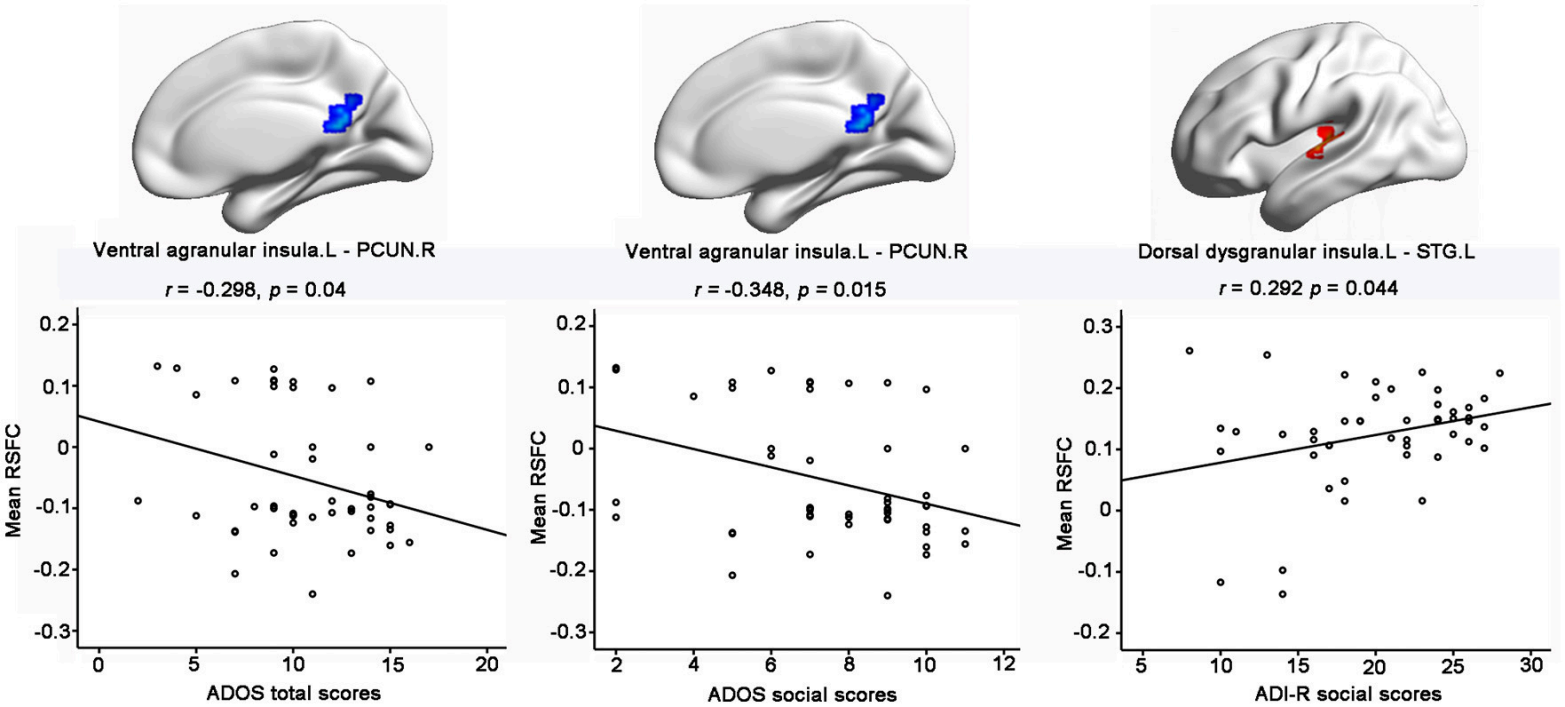
Significant correlations between RSFC of insular subregions and clinical characteristics of ASD with the age, gender, handedness, verbal IQs, performance IQs, and full-scale IQs as covariates. Significance was determined with a threshold of *p* < 0.05.

Importantly, significantly partial correlations between the RSFC of the insular subregions and the clinical characteristics of the children with ASD were identified with age, gender, handedness, full-scale IQs, verbal IQs, and performance IQs as covariates (Figure 3). The hypo-connectivity between the left ventral agranular insula and PCUN.R was negatively correlated with the ADOS total/social scores in the ASD group. Moreover, the hyper-connectivity between the left dorsal dysgranular insula and STG.L was positively correlated with the ADI-R social scores in the ASD group.

## Discussion

In the present study, we investigated the distinct functional alterations of the insular subregions between the children with ASD and TD groups. Compared with the TD group, the ASD group showed different connectivity patterns in the left ventral agranular insula, right ventral dysgranular and granular insula, and left dorsal dysgranular insula, characterized by hypo-connectivity and/or hyper-connectivity with specific brain regions. Furthermore, both the hypo-connectivity and hyper-connectivity of the insular subregions were significantly associated with the core symptoms of ASD.

ASD is a complex neurodevelopmental disorder that affects multiple cognitive domains. Recent theoretical models have highlighted the need to consider ASD as a disorder associated with several large-scale networks (Belmonte et al., 2004; Welchew et al., 2005; Geschwind and Levitt, 2007). Using independent component analysis, Uddin et al. demonstrated that compared with TD children, children with ASD exhibited altered functional connectivity of the salience network, DMN, frontotemporal network, motor network, and visual network (Uddin et al., 2013b). Our results support this notion by demonstrating hyper-connectivity and/or hypo-connectivity of the insular subregions involved in different brain networks, supporting several behavioral domains known to be impaired in the complex symptoms of the ASD.

Specifically, hypo-connectivity was observed between the left ventral agranular insula and bilateral PCUN in the ASD group. The left ventral agranular insula is mainly located in the left anterior insula, which is a hub in the salience network. Previous studies demonstrated that the anterior insula plays a critical role in processing information relevant to social functioning as a sort of “hub” that mediates interactions between the DMN and the central-executive networks (Sridharan et al., 2008; Uddin and Menon, 2009; Menon and Uddin, 2010). Apart from the key part of the DMN, the precuneus has a role in emotion, self-referential thinking, and projection processes critical for social development (Cavanna and Trimble, 2006) and has also been linked to atypical mentalizing or theory of mind in ASD (Castelli et al., 2002; Wang et al., 2007). Given the crucial role of both the anterior insula and the precuneus in some aspects of social cognition, the significant hypo-connectivity between the left ventral agranular insula and bilateral precuneus might contribute to social interaction deficits in ASD. Moreover, this suggestion was further supported by our result, which showed negative correlation between the hypo-connectivity of the left ventral agranular insula—PCUN.R with the ADOS total/social scores in the ASD group.

In addition, compared with the TD group, the ASD group showed hypo-connectivity between the right ventral dysgranular and granular insula and SMG.R. According to the behavioral results of the Brainnetome Atlas, the right ventral dysgranular and granular insula is mostly associated with emotion. The right insula is associated with sympathetic (“aroused”) functions based on anatomical evidence of left-to-right asymmetry in peripheral autonomic efferent neurons and homeostatic afferent neurons, as well as a review of neuroimaging literature (Yamada et al., 2016). Moreover, SMG.R has been consistently shown to play a crucial role in emotion processing (Singer et al., 2009; Lamm et al., 2011). Since there is indeed good evidence that empathy may be impaired in ASD (Baron-Cohen and Wheelwright, 2004; Jones et al., 2010; Lockwood et al., 2013), it is reasonable to conclude that the hypo-connectivity between the right ventral dysgranular and granular insula and SMG.R might contribute to the deficits of emotion in ASD.

Furthermore, hypo-connectivity between the left dorsal dysgranular insula and the right cuneus was also identified in the ASD. According to the behavioral results of the Brainnetome Atlas, both the left dorsal dysgranular insula and the right cuneus are mostly associated with perception, particularly the visual processing. When engaged in visual processing, ASD often exhibit enhanced perceptual abilities with more activity in the occipital regions, such as visual search (Keehn et al., 2008; Joseph et al., 2009; Samson et al., 2012) and visual discrimination (Bertone et al., 2005). However, a fMRI study of visual search (Keehn et al., 2013) is inconsistent with previous studies by showing neither group differences of any behavioral search measures nor differential patterns of activation in ASD. Due to these contradictory results and lack of visual measurements in our research, further investigation is needed to explain the result of the hypo-connectivity between the left dorsal dysgranular insula and the right cuneus in the ASD.

In addition, significant hyper-connectivity was identified between the left dorsal dysgranular insula and the STG.L in ASD. In line with the hyper-connectivity of our result, previous studies showed increased activity during social reward learning (Choi et al., 2015), sentence comprehension task (Just et al., 2004), and facial emotion processing (Dalton et al., 2008), as well as increased gray matter volume in STG.L in ASD (Waiter et al., 2004). Moreover, our finding of functional hyper-connectivity is also supported by the positive correlation with the ADI-R social scores, which implied that children with greater connectivity exhibited more severe impairment in the social domain. Considering that the left dorsal dysgranular insula is mostly associated with perception according to the behavioral results of the Brainnetome Atlas, this brain–behavior relationship suggests that aberrant functional connectivity may underlie the deficits of social perception in the ASD. Notably, the relationship between functional connectivity abnormalities of the left dorsal dysgranular insula and social severity was limited to the ADI-R social score, which is based on early social development, but not the ADOS score, which is a complimentary measure that rates current social functioning (Kleinhans et al., 2008). This pattern of correlation may imply that early development history plays an important part in the hyper-connectivity between the left dorsal dysgranular insula and the STG.L in ASD. Moreover, our finding is relatively novel. Further studies with large samples are needed to confirm this association and investigate its causes and clinical implications.

Several limitations should be acknowledged in our current study. First, some of the patients with ASD were given one or more psychotropic medications. Studies of drug-naïve patients to exclude the effects of medication on our findings are warranted. Second, given the high possibility that the RSFC can be effected by age (Dosenbach et al., 2010), we involved only children with a narrow range of age. However, this restriction left us no chance to address the developmental effects in the ASD. Thus, it remains a crucial topic for further investigation on the interaction between developmental changes and alterations of the RSFC in ASD.

In conclusion, compared with the TD group, the ASD group showed different connectivity patterns in the left ventral agranular insula, right ventral dysgranular and granular insula, and left dorsal dysgranular insula, characterized by significant hyper-connectivity and/or hypo-connectivity with specific brain regions. Furthermore, both the hypo-connectivity and hyper-connectivity of the insular subregions were significantly associated with the severity of ASD in children. Our research demonstrated distinct abnormalities in the RSFC patterns of the insular subregions and emphasized the importance of the subdivisions within the insula to potentially impact the functional difference in children with ASD. Moreover, these results might help us to better understand the mechanisms underlying the symptoms in children with ASD and might elucidate potential biomarkers for clinical applications.

## Author contributions

HW and ZhZ acquired the data. JX, ZX, TL, and ZfZ analyzed data. JX, QH, LZ, and YG conceived this study and designed experiments. JX wrote the article with help of TL and ZfZ. All authors were involved in data interpretation and critically revising the manuscript.

### Conflict of interest statement

The authors declare that the research was conducted in the absence of any commercial or financial relationships that could be construed as a potential conflict of interest.
